# Dichloridobis(4-chloro­benz­yl)(4,4′-dimethyl-2,2′-bipyridine-κ^2^ *N*,*N*′)tin(IV)

**DOI:** 10.1107/S1600536809049381

**Published:** 2009-11-25

**Authors:** Thy Chun Keng, Kong Mun Lo, Seik Weng Ng

**Affiliations:** a Department of Chemistry, University of Malaya, 50603 Kuala Lumpur, Malaysia

## Abstract

The Sn^IV^ atom in the title compound, [Sn(C_7_H_6_Cl)_2_Cl_2_(C_12_H_12_N_2_)], is coordinated by the bidentate *N*-heterocycle mol­ecule, two chloro­benzyl anions and two Cl^−^ anions in a distorted *trans*-C_2_SnCl_2_N_2_ octa­hedral geometry [C—Sn—C = 178.4 (1)°]. In the mol­ecular structure, the two benzene rings are oriented at a dihedral angle of 39.62 (17)°.

## Related literature

For the synthesis of bis­(4-chloro­benz­yl)tin dichloride, see: Sisido *et al.* (1961 ▶). For the 1,10-phenanthroline adduct, see: Tan *et al.* (2009 ▶).
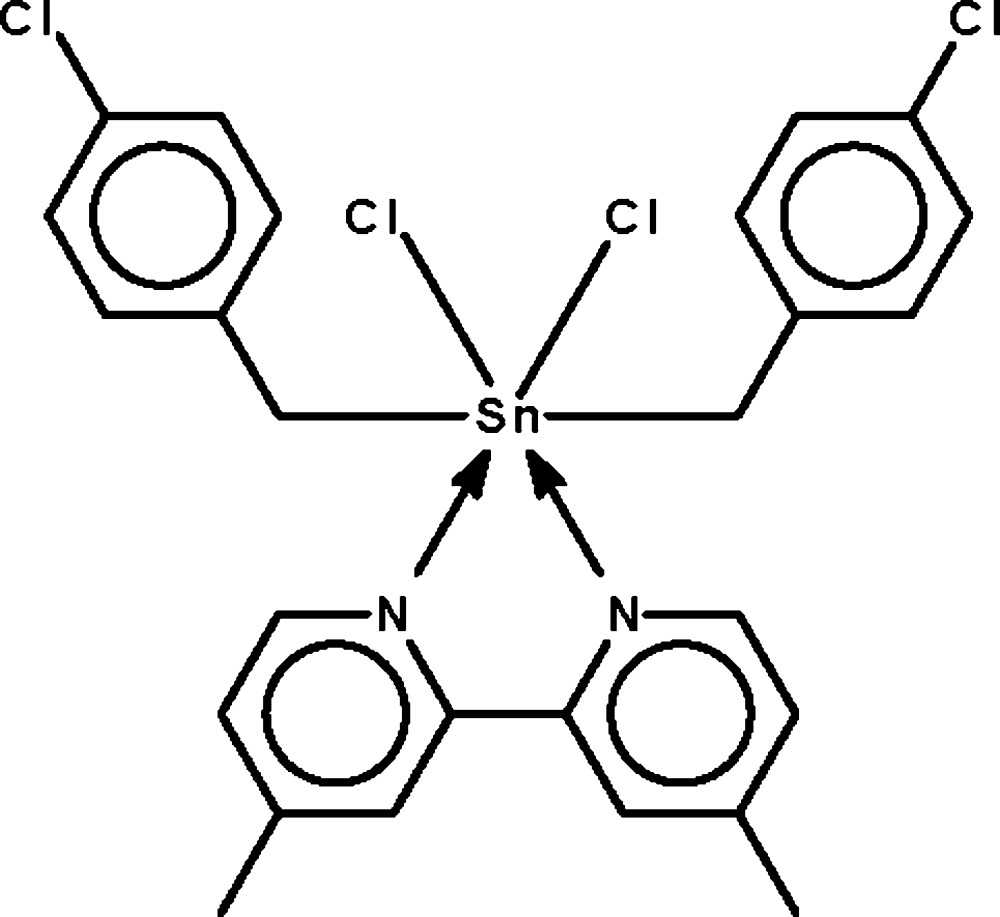


## Experimental

### 

#### Crystal data

[Sn(C_7_H_6_Cl)_2_Cl_2_(C_12_H_12_N_2_)]
*M* *_r_* = 624.96
Monoclinic, 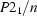

*a* = 11.4035 (6) Å
*b* = 14.6804 (8) Å
*c* = 16.9270 (9) Å
β = 101.9182 (9)°
*V* = 2772.6 (3) Å^3^
*Z* = 4
Mo *K*α radiation
μ = 1.32 mm^−1^
*T* = 293 K
0.30 × 0.28 × 0.20 mm

#### Data collection

Bruker SMART APEX diffractometer
Absorption correction: multi-scan (*SADABS*; Sheldrick, 1996 ▶) *T*_min_ = 0.692, *T*_max_ = 0.778
15766 measured reflections
6263 independent reflections
4883 reflections with *I* > 2σ(*I*)
*R*_int_ = 0.017

#### Refinement

*R*[*F* ^2^ > 2σ(*F* ^2^)] = 0.031
*wR*(*F* ^2^) = 0.095
*S* = 1.00
6263 reflections
300 parameters
H-atom parameters constrained
Δρ_max_ = 0.78 e Å^−3^
Δρ_min_ = −0.49 e Å^−3^

### 

Data collection: *APEX2* (Bruker, 2008 ▶); cell refinement: *SAINT* (Bruker, 2008 ▶); data reduction: *SAINT*; program(s) used to solve structure: *SHELXS97* (Sheldrick, 2008 ▶); program(s) used to refine structure: *SHELXL97* (Sheldrick, 2008 ▶); molecular graphics: *X-SEED* (Barbour, 2001 ▶); software used to prepare material for publication: *publCIF* (Westrip, 2009 ▶).

## Supplementary Material

Crystal structure: contains datablocks global, I. DOI: 10.1107/S1600536809049381/xu2682sup1.cif

Structure factors: contains datablocks I. DOI: 10.1107/S1600536809049381/xu2682Isup2.hkl

Additional supplementary materials:  crystallographic information; 3D view; checkCIF report

## Figures and Tables

**Fig. 1.**
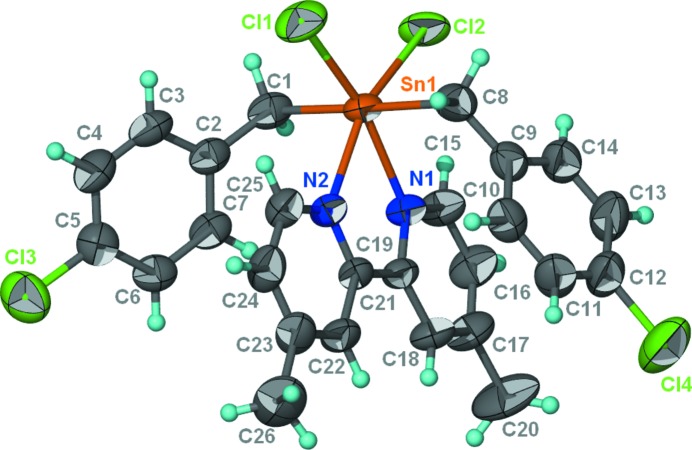
Thermal ellipsoid plot (Barbour, 2001) of SnCl2(C10H12N2)(C7H6Cl)2 at the 50% probability level; hydrogen atoms are drawn as spheres of arbitrary radius.

## supplementary crystallographic information

### Experimental

Di(4-chlorobenzyl)tin dichloride was synthesized by the reaction of 4-chlorobenzyl chloride and metallic tin (Sisido *et al.*). The diorganotin compound (0.44 g, 1 mmol) and 4,4'-dimethyl-2,2'-bipyridine (0.18 g, 1 mmol) was heated in chloroform (30 ml) for 1 h. Faint-yellow crystals separated upon slow evaporation of the solvent.

### Refinement

Carbon-bound H-atoms were placed in calculated positions (C–H 0.93–0.97 Å) and were included in the refinement in the riding model approximation, with *U*(H) set to 1.2–1.5*U*(C).

### Crystal data

**Table:**

[Sn(C_7_H_6_Cl)_2_Cl_2_(C_12_H_12_N_2_)]	*F*(000) = 1248
*M**_r_* = 624.96	*D*_x_ = 1.497 Mg m^−^^3^
Monoclinic, *P*2_1_/*n*	Mo *K*α radiation, λ = 0.71073 Å
Hall symbol: -P 2yn	Cell parameters from 6177 reflections
*a* = 11.4035 (6) Å	θ = 2.4–28.3°
*b* = 14.6804 (8) Å	µ = 1.32 mm^−^^1^
*c* = 16.9270 (9) Å	*T* = 293 K
β = 101.9182 (9)°	Block, pale yellow
*V* = 2772.6 (3) Å^3^	0.30 × 0.28 × 0.20 mm
*Z* = 4	

### Data collection

**Table:**

Bruker SMART APEX diffractometer	6263 independent reflections
Radiation source: fine-focus sealed tube	4883 reflections with *I* > 2σ(*I*)
graphite	*R*_int_ = 0.017
ω scans	θ_max_ = 27.5°, θ_min_ = 1.9°
Absorption correction: multi-scan (*SADABS*; Sheldrick, 1996)	*h* = −14→14
*T*_min_ = 0.692, *T*_max_ = 0.778	*k* = −19→18
15766 measured reflections	*l* = −21→17

### Refinement

**Table:**

Refinement on *F*^2^	Primary atom site location: structure-invariant direct methods
Least-squares matrix: full	Secondary atom site location: difference Fourier map
*R*[*F*^2^ > 2σ(*F*^2^)] = 0.031	Hydrogen site location: inferred from neighbouring sites
*wR*(*F*^2^) = 0.095	H-atom parameters constrained
*S* = 1.00	*w* = 1/[σ^2^(*F*_o_^2^) + (0.0523*P*)^2^ + 1.1119*P*] where *P* = (*F*_o_^2^ + 2*F*_c_^2^)/3
6263 reflections	(Δ/σ)_max_ = 0.001
300 parameters	Δρ_max_ = 0.78 e Å^−^^3^
0 restraints	Δρ_min_ = −0.49 e Å^−^^3^

### Fractional atomic coordinates and isotropic or equivalent isotropic displacement parameters (Å^2^)

**Table:**

	*x*	*y*	*z*	*U*_iso_*/*U*_eq_	
Sn1	0.278374 (15)	0.231209 (14)	0.536683 (11)	0.05372 (8)	
Cl1	0.06174 (7)	0.19394 (9)	0.52457 (7)	0.0993 (3)	
Cl2	0.28579 (8)	0.31620 (8)	0.40783 (5)	0.0894 (3)	
Cl3	0.35723 (13)	−0.23372 (9)	0.67467 (9)	0.1119 (4)	
Cl4	0.68248 (17)	0.53534 (12)	0.79043 (9)	0.1696 (8)	
N1	0.4862 (2)	0.24492 (17)	0.57811 (15)	0.0558 (5)	
N2	0.34094 (19)	0.16803 (15)	0.66356 (12)	0.0513 (5)	
C1	0.3049 (3)	0.1063 (2)	0.47620 (17)	0.0686 (8)	
H1A	0.2382	0.0985	0.4309	0.082*	
H1B	0.3768	0.1127	0.4545	0.082*	
C2	0.3163 (3)	0.0226 (2)	0.52556 (17)	0.0606 (7)	
C3	0.2208 (3)	−0.0357 (2)	0.5251 (2)	0.0721 (8)	
H3	0.1468	−0.0219	0.4926	0.087*	
C4	0.2322 (3)	−0.1133 (3)	0.5713 (2)	0.0788 (9)	
H4	0.1670	−0.1518	0.5696	0.095*	
C5	0.3412 (3)	−0.1333 (2)	0.6199 (2)	0.0726 (8)	
C6	0.4370 (3)	−0.0777 (2)	0.62247 (19)	0.0680 (8)	
H6	0.5105	−0.0920	0.6555	0.082*	
C7	0.4249 (3)	−0.0004 (2)	0.57607 (18)	0.0639 (7)	
H7	0.4907	0.0377	0.5784	0.077*	
C8	0.2463 (3)	0.3566 (2)	0.5963 (2)	0.0728 (8)	
H8A	0.2064	0.3988	0.5553	0.087*	
H8B	0.1911	0.3434	0.6313	0.087*	
C9	0.3519 (3)	0.4035 (2)	0.64484 (19)	0.0664 (8)	
C10	0.3852 (3)	0.3904 (2)	0.7278 (2)	0.0735 (8)	
H10	0.3389	0.3529	0.7535	0.088*	
C11	0.4853 (4)	0.4318 (3)	0.7727 (2)	0.0858 (11)	
H11	0.5059	0.4230	0.8282	0.103*	
C12	0.5538 (4)	0.4856 (3)	0.7352 (2)	0.0943 (12)	
C13	0.5227 (4)	0.5011 (3)	0.6537 (2)	0.0985 (13)	
H13	0.5691	0.5394	0.6288	0.118*	
C14	0.4235 (4)	0.4599 (2)	0.6093 (2)	0.0797 (9)	
H14	0.4035	0.4699	0.5539	0.096*	
C15	0.5562 (3)	0.2800 (2)	0.5314 (2)	0.0705 (9)	
H15	0.5227	0.2934	0.4778	0.085*	
C16	0.6750 (3)	0.2967 (3)	0.5597 (2)	0.0813 (10)	
H16	0.7213	0.3205	0.5254	0.098*	
C17	0.7262 (3)	0.2785 (3)	0.6385 (3)	0.0825 (10)	
C18	0.6537 (3)	0.2412 (2)	0.6866 (2)	0.0689 (8)	
H18	0.6858	0.2277	0.7404	0.083*	
C19	0.5348 (2)	0.22410 (18)	0.65515 (16)	0.0516 (6)	
C20	0.8561 (4)	0.2981 (5)	0.6727 (3)	0.139 (2)	
H20A	0.8984	0.3037	0.6295	0.209*	
H20B	0.8899	0.2492	0.7077	0.209*	
H20C	0.8629	0.3540	0.7028	0.209*	
C21	0.4549 (2)	0.17973 (18)	0.70150 (15)	0.0528 (6)	
C22	0.4934 (3)	0.1484 (2)	0.77971 (18)	0.0733 (9)	
H22	0.5725	0.1579	0.8060	0.088*	
C23	0.4162 (4)	0.1035 (3)	0.81914 (19)	0.0831 (10)	
C24	0.3023 (3)	0.0899 (2)	0.7781 (2)	0.0772 (9)	
H24	0.2483	0.0580	0.8019	0.093*	
C25	0.2669 (3)	0.1234 (2)	0.70125 (18)	0.0632 (7)	
H25	0.1879	0.1147	0.6744	0.076*	
C26	0.4588 (5)	0.0702 (4)	0.9050 (2)	0.138 (2)	
H26A	0.4559	0.0049	0.9060	0.206*	
H26B	0.4079	0.0946	0.9385	0.206*	
H26C	0.5397	0.0902	0.9249	0.206*	

### Atomic displacement parameters (Å^2^)

**Table:**

	*U*^11^	*U*^22^	*U*^33^	*U*^12^	*U*^13^	*U*^23^
Sn1	0.04228 (11)	0.06803 (14)	0.04599 (12)	0.00021 (8)	−0.00217 (8)	0.00089 (8)
Cl1	0.0438 (4)	0.1234 (8)	0.1241 (8)	−0.0074 (4)	0.0018 (4)	−0.0088 (7)
Cl2	0.0821 (6)	0.1201 (8)	0.0552 (5)	−0.0123 (5)	−0.0105 (4)	0.0282 (5)
Cl3	0.1097 (9)	0.1036 (8)	0.1256 (10)	−0.0010 (6)	0.0316 (8)	0.0320 (7)
Cl4	0.2037 (16)	0.1603 (14)	0.1127 (10)	−0.1049 (13)	−0.0418 (10)	0.0234 (9)
N1	0.0450 (12)	0.0726 (15)	0.0471 (13)	−0.0018 (10)	0.0032 (10)	0.0021 (10)
N2	0.0498 (11)	0.0597 (13)	0.0425 (11)	−0.0029 (9)	0.0052 (9)	−0.0037 (10)
C1	0.0699 (18)	0.087 (2)	0.0462 (15)	0.0005 (16)	0.0043 (13)	−0.0122 (15)
C2	0.0583 (15)	0.0721 (19)	0.0503 (15)	0.0011 (13)	0.0084 (12)	−0.0151 (13)
C3	0.0534 (17)	0.088 (2)	0.0686 (19)	−0.0050 (15)	−0.0018 (14)	−0.0151 (18)
C4	0.0645 (19)	0.085 (2)	0.086 (2)	−0.0156 (17)	0.0133 (17)	−0.012 (2)
C5	0.074 (2)	0.076 (2)	0.071 (2)	−0.0009 (16)	0.0231 (16)	−0.0028 (17)
C6	0.0580 (17)	0.082 (2)	0.0625 (18)	0.0071 (15)	0.0097 (14)	−0.0047 (16)
C7	0.0520 (15)	0.079 (2)	0.0605 (17)	−0.0022 (13)	0.0127 (13)	−0.0135 (15)
C8	0.0664 (18)	0.072 (2)	0.077 (2)	0.0131 (15)	0.0066 (16)	−0.0032 (17)
C9	0.078 (2)	0.0556 (16)	0.0646 (18)	0.0083 (14)	0.0125 (15)	−0.0035 (14)
C10	0.094 (2)	0.0649 (19)	0.0637 (19)	0.0016 (17)	0.0216 (17)	−0.0017 (15)
C11	0.123 (3)	0.074 (2)	0.0553 (18)	−0.008 (2)	0.0051 (19)	0.0007 (16)
C12	0.123 (3)	0.076 (2)	0.073 (2)	−0.032 (2)	−0.005 (2)	0.0002 (19)
C13	0.133 (4)	0.079 (2)	0.079 (2)	−0.038 (2)	0.012 (2)	0.009 (2)
C14	0.104 (3)	0.069 (2)	0.0620 (19)	−0.0090 (19)	0.0083 (18)	0.0058 (16)
C15	0.0569 (17)	0.100 (3)	0.0549 (17)	−0.0075 (15)	0.0106 (14)	0.0061 (16)
C16	0.0580 (18)	0.111 (3)	0.079 (2)	−0.0180 (18)	0.0228 (17)	−0.002 (2)
C17	0.0478 (17)	0.110 (3)	0.085 (3)	−0.0116 (16)	0.0041 (16)	−0.013 (2)
C18	0.0496 (16)	0.091 (2)	0.0591 (18)	−0.0032 (14)	−0.0051 (13)	−0.0041 (16)
C19	0.0464 (13)	0.0581 (15)	0.0456 (14)	0.0008 (11)	−0.0012 (11)	−0.0047 (11)
C20	0.054 (2)	0.222 (6)	0.133 (4)	−0.037 (3)	−0.001 (2)	−0.004 (4)
C21	0.0567 (14)	0.0535 (15)	0.0434 (13)	−0.0033 (11)	−0.0006 (11)	−0.0032 (11)
C22	0.080 (2)	0.076 (2)	0.0520 (17)	−0.0178 (16)	−0.0151 (14)	0.0107 (15)
C23	0.112 (3)	0.079 (2)	0.0510 (17)	−0.026 (2)	−0.0015 (18)	0.0113 (16)
C24	0.101 (3)	0.074 (2)	0.0589 (18)	−0.0274 (18)	0.0211 (18)	−0.0006 (16)
C25	0.0643 (17)	0.0681 (18)	0.0575 (17)	−0.0124 (14)	0.0131 (13)	−0.0064 (14)
C26	0.184 (5)	0.145 (5)	0.066 (2)	−0.062 (4)	−0.016 (3)	0.040 (3)

### Geometric parameters (Å, °)

**Table:**

Sn1—C1	2.151 (3)	C10—H10	0.9300
Sn1—C8	2.166 (3)	C11—C12	1.357 (5)
Sn1—N2	2.313 (2)	C11—H11	0.9300
Sn1—N1	2.337 (2)	C12—C13	1.371 (5)
Sn1—Cl1	2.4970 (9)	C13—C14	1.363 (5)
Sn1—Cl2	2.5293 (8)	C13—H13	0.9300
Cl3—C5	1.731 (4)	C14—H14	0.9300
Cl4—C12	1.730 (4)	C15—C16	1.363 (5)
N1—C19	1.343 (4)	C15—H15	0.9300
N1—C15	1.337 (4)	C16—C17	1.366 (5)
N2—C25	1.331 (4)	C16—H16	0.9300
N2—C21	1.336 (3)	C17—C18	1.388 (5)
C1—C2	1.476 (5)	C17—C20	1.502 (5)
C1—H1A	0.9700	C18—C19	1.374 (4)
C1—H1B	0.9700	C18—H18	0.9300
C2—C3	1.384 (4)	C19—C21	1.472 (4)
C2—C7	1.394 (4)	C20—H20A	0.9600
C3—C4	1.372 (5)	C20—H20B	0.9600
C3—H3	0.9300	C20—H20C	0.9600
C4—C5	1.374 (5)	C21—C22	1.384 (4)
C4—H4	0.9300	C22—C23	1.378 (5)
C5—C6	1.358 (5)	C22—H22	0.9300
C6—C7	1.371 (5)	C23—C24	1.356 (5)
C6—H6	0.9300	C23—C26	1.515 (5)
C7—H7	0.9300	C24—C25	1.371 (4)
C8—C9	1.480 (5)	C24—H24	0.9300
C8—H8A	0.9700	C25—H25	0.9300
C8—H8B	0.9700	C26—H26A	0.9600
C9—C10	1.391 (5)	C26—H26B	0.9600
C9—C14	1.385 (5)	C26—H26C	0.9600
C10—C11	1.375 (5)		
			
C1—Sn1—C8	178.42 (12)	C9—C10—H10	119.4
C1—Sn1—N2	93.06 (10)	C12—C11—C10	119.4 (3)
C8—Sn1—N2	87.58 (11)	C12—C11—H11	120.3
C1—Sn1—N1	88.99 (11)	C10—C11—H11	120.3
C8—Sn1—N1	92.59 (11)	C11—C12—C13	120.9 (4)
N2—Sn1—N1	69.92 (8)	C11—C12—Cl4	119.9 (3)
C1—Sn1—Cl1	90.48 (9)	C13—C12—Cl4	119.2 (3)
C8—Sn1—Cl1	88.01 (9)	C14—C13—C12	119.5 (4)
N2—Sn1—Cl1	95.66 (6)	C14—C13—H13	120.2
N1—Sn1—Cl1	165.52 (7)	C12—C13—H13	120.2
C1—Sn1—Cl2	88.68 (9)	C13—C14—C9	121.5 (3)
C8—Sn1—Cl2	91.21 (10)	C13—C14—H14	119.2
N2—Sn1—Cl2	160.01 (6)	C9—C14—H14	119.2
N1—Sn1—Cl2	90.22 (6)	N1—C15—C16	122.2 (3)
Cl1—Sn1—Cl2	104.24 (4)	N1—C15—H15	118.9
C19—N1—C15	119.0 (3)	C16—C15—H15	118.9
C19—N1—Sn1	117.50 (18)	C15—C16—C17	120.1 (3)
C15—N1—Sn1	123.2 (2)	C15—C16—H16	120.0
C25—N2—C21	118.9 (2)	C17—C16—H16	120.0
C25—N2—Sn1	122.54 (19)	C16—C17—C18	117.6 (3)
C21—N2—Sn1	118.53 (17)	C16—C17—C20	121.8 (4)
C2—C1—Sn1	116.38 (19)	C18—C17—C20	120.5 (4)
C2—C1—H1A	108.2	C19—C18—C17	120.3 (3)
Sn1—C1—H1A	108.2	C19—C18—H18	119.9
C2—C1—H1B	108.2	C17—C18—H18	119.9
Sn1—C1—H1B	108.2	N1—C19—C18	120.8 (3)
H1A—C1—H1B	107.3	N1—C19—C21	116.2 (2)
C3—C2—C7	116.8 (3)	C18—C19—C21	123.0 (3)
C3—C2—C1	122.4 (3)	C17—C20—H20A	109.5
C7—C2—C1	120.8 (3)	C17—C20—H20B	109.5
C4—C3—C2	122.0 (3)	H20A—C20—H20B	109.5
C4—C3—H3	119.0	C17—C20—H20C	109.5
C2—C3—H3	119.0	H20A—C20—H20C	109.5
C3—C4—C5	119.1 (3)	H20B—C20—H20C	109.5
C3—C4—H4	120.4	N2—C21—C22	120.1 (3)
C5—C4—H4	120.4	N2—C21—C19	116.6 (2)
C6—C5—C4	120.9 (3)	C22—C21—C19	123.2 (3)
C6—C5—Cl3	120.0 (3)	C23—C22—C21	120.9 (3)
C4—C5—Cl3	119.1 (3)	C23—C22—H22	119.5
C5—C6—C7	119.6 (3)	C21—C22—H22	119.5
C5—C6—H6	120.2	C24—C23—C22	117.5 (3)
C7—C6—H6	120.2	C24—C23—C26	121.9 (4)
C6—C7—C2	121.6 (3)	C22—C23—C26	120.6 (4)
C6—C7—H7	119.2	C23—C24—C25	119.8 (3)
C2—C7—H7	119.2	C23—C24—H24	120.1
C9—C8—Sn1	117.3 (2)	C25—C24—H24	120.1
C9—C8—H8A	108.0	N2—C25—C24	122.7 (3)
Sn1—C8—H8A	108.0	N2—C25—H25	118.7
C9—C8—H8B	108.0	C24—C25—H25	118.7
Sn1—C8—H8B	108.0	C23—C26—H26A	109.5
H8A—C8—H8B	107.2	C23—C26—H26B	109.5
C10—C9—C14	117.3 (3)	H26A—C26—H26B	109.5
C10—C9—C8	121.2 (3)	C23—C26—H26C	109.5
C14—C9—C8	121.4 (3)	H26A—C26—H26C	109.5
C11—C10—C9	121.3 (3)	H26B—C26—H26C	109.5
C11—C10—H10	119.4		
			
C1—Sn1—N1—C15	82.9 (3)	Sn1—C8—C9—C10	−95.9 (3)
N2—Sn1—N1—C15	176.5 (3)	C14—C9—C10—C11	−0.1 (5)
Cl1—Sn1—N1—C15	170.9 (2)	C8—C9—C10—C11	178.0 (3)
Cl2—Sn1—N1—C15	−5.8 (3)	C9—C10—C11—C12	−0.8 (6)
C1—Sn1—N1—C19	−103.4 (2)	C10—C11—C12—C13	1.7 (7)
C8—Sn1—N1—C19	76.7 (2)	C10—C11—C12—Cl4	−178.0 (3)
N2—Sn1—N1—C19	−9.79 (19)	C11—C12—C13—C14	−1.8 (7)
Cl1—Sn1—N1—C19	−15.4 (4)	Cl4—C12—C13—C14	178.0 (4)
Cl2—Sn1—N1—C19	167.9 (2)	C12—C13—C14—C9	0.9 (7)
C1—Sn1—N2—C25	−86.3 (2)	C10—C9—C14—C13	0.0 (6)
C8—Sn1—N2—C25	92.2 (2)	C8—C9—C14—C13	−178.1 (4)
N1—Sn1—N2—C25	−174.1 (2)	C19—N1—C15—C16	−1.1 (5)
Cl1—Sn1—N2—C25	4.5 (2)	Sn1—N1—C15—C16	172.5 (3)
Cl2—Sn1—N2—C25	179.13 (18)	N1—C15—C16—C17	−0.7 (6)
C1—Sn1—N2—C21	96.2 (2)	C15—C16—C17—C18	1.4 (6)
C8—Sn1—N2—C21	−85.3 (2)	C15—C16—C17—C20	−178.4 (4)
N1—Sn1—N2—C21	8.36 (19)	C16—C17—C18—C19	−0.3 (6)
Cl1—Sn1—N2—C21	−173.05 (19)	C20—C17—C18—C19	179.5 (4)
Cl2—Sn1—N2—C21	1.6 (3)	C15—N1—C19—C18	2.2 (4)
C8—Sn1—C1—C2	−97 (5)	Sn1—N1—C19—C18	−171.8 (2)
N2—Sn1—C1—C2	16.6 (2)	C15—N1—C19—C21	−175.8 (3)
N1—Sn1—C1—C2	86.5 (2)	Sn1—N1—C19—C21	10.3 (3)
Cl1—Sn1—C1—C2	−79.0 (2)	C17—C18—C19—N1	−1.5 (5)
Cl2—Sn1—C1—C2	176.7 (2)	C17—C18—C19—C21	176.3 (3)
Sn1—C1—C2—C3	97.9 (3)	C25—N2—C21—C22	−2.2 (4)
Sn1—C1—C2—C7	−81.2 (3)	Sn1—N2—C21—C22	175.5 (2)
C7—C2—C3—C4	−0.8 (5)	C25—N2—C21—C19	176.1 (2)
C1—C2—C3—C4	−179.9 (3)	Sn1—N2—C21—C19	−6.3 (3)
C2—C3—C4—C5	0.6 (5)	N1—C19—C21—N2	−2.7 (4)
C3—C4—C5—C6	−0.3 (5)	C18—C19—C21—N2	179.4 (3)
C3—C4—C5—Cl3	−177.1 (3)	N1—C19—C21—C22	175.5 (3)
C4—C5—C6—C7	0.3 (5)	C18—C19—C21—C22	−2.4 (5)
Cl3—C5—C6—C7	177.0 (2)	N2—C21—C22—C23	1.2 (5)
C5—C6—C7—C2	−0.5 (5)	C19—C21—C22—C23	−176.9 (3)
C3—C2—C7—C6	0.7 (4)	C21—C22—C23—C24	1.0 (6)
C1—C2—C7—C6	179.9 (3)	C21—C22—C23—C26	−179.4 (4)
N2—Sn1—C8—C9	65.4 (3)	C22—C23—C24—C25	−2.2 (6)
N1—Sn1—C8—C9	−4.4 (3)	C26—C23—C24—C25	178.2 (4)
Cl1—Sn1—C8—C9	161.1 (3)	C21—N2—C25—C24	0.9 (4)
Cl2—Sn1—C8—C9	−94.6 (3)	Sn1—N2—C25—C24	−176.6 (2)
Sn1—C8—C9—C14	82.1 (4)	C23—C24—C25—N2	1.4 (5)
